# Supplementary material for: Does Methionine Status Influence the Outcome of Selenomethinione Supplementation? A Comparative Study of Metabolic and Selenium Levels in HepG2 Cells
Source: Nutrients. 2022 Sep 8;14(18):3705. doi: 10.3390/nu14183705 (PMC9506159; doi:10.3390/nu14183705)
Supplement: Supplementary file 1 [file nutrients-14-03705-s001.zip › nutrients-1857687-supplementary.pdf]

Figure S1. The relative amounts of viable cells in the 96-well plates that containing different concentrations of Met and SeMet after 48 h incubation.

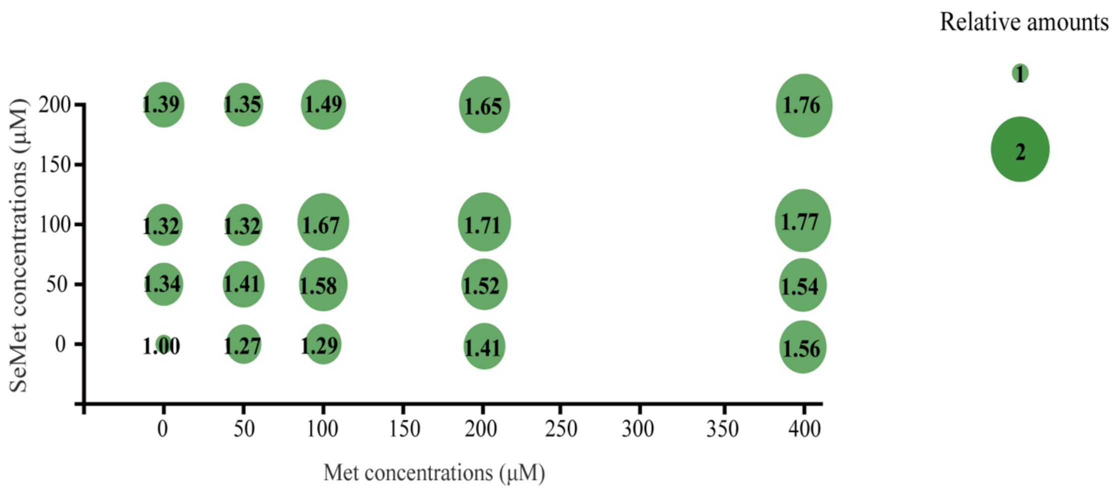

Figure S2. 2D NMR spectra of HepG2 cell extracts: (A) An overlap spectrum of ^{1}H-^{13}C HSQC (purple), ^{1}H-^{13}C HMBC (blue) and ^{1}H spectroscopy (red); (B) An overlap spectrum of ^{1}H-^{1}H COSY (purple), ^{1}H-^{1}H TOCSY (blue) and ^{1}H spectroscopy (red).

A

B

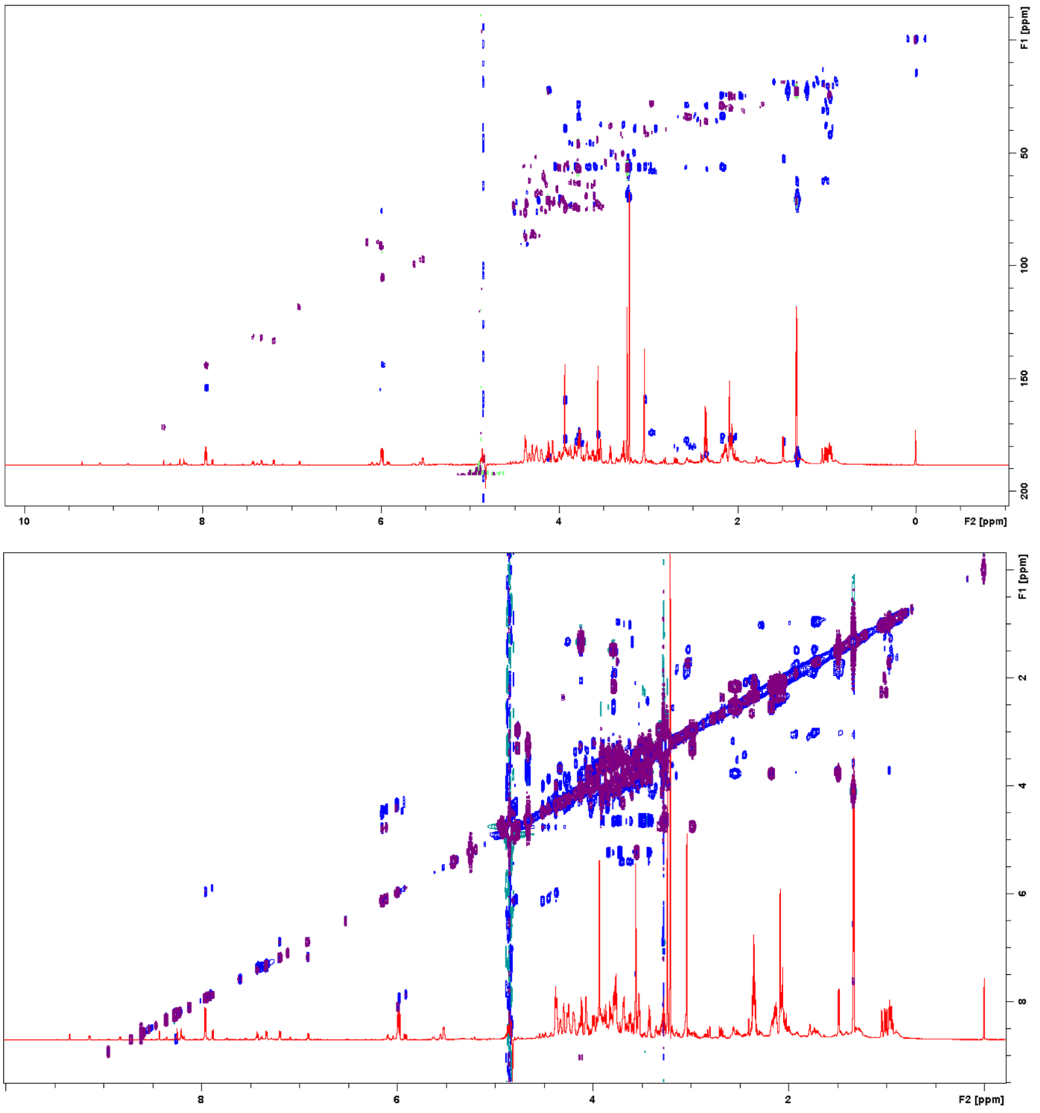

Table S1. Primer sequences for RT-qPCR analysis.

Genes	Forward primer	Reverse primer
GPX1	5′- CAGTCGGTGTATGCCTTCTCG-3′	5′- GAGGGACGCCACATTCTCG-3′
GPX2	5′- GAATGGGCAGAACGAGCATC-3′	5′- CCGGCCCTATGAGGAACTTC-3′
GPX4	5′- GAGGCAAGACCGAAGTAAACTAC-3′	5′- CCGAACTGGTTACACGGGAA-3′
TXNRD1	5′- TAGGACAAGCCCTGCAAGACT-3′	5′- CCCCAATTCAAAGAGCCAATGT-3′
TXNRD2	5′- CTAGCCCCGACACTCAGAAGA- 3′	5′-GGCCATGATCGCTATGGGT- 3′
SELENOP	5′- AACTGCTCTCTCACGACTCTC -3′	5′- AGCATTTGGTGCTCCTGGTT -3′
GAPDH	5′- GGCATCCTGGGCTACACTGA -3′	5′- GGAGTGGGTGTCGCTGTTG -3′

Table S2. NMR data for the metabolites assignment in HepG2 cell extracts.

No.	Metabolites	Group	δ^1H (multiplicity[a])	δ^{13}C
1	Acetate	CH_3	1.93(s)	26.4
		COOH	—	184.0
2	Alanine (Ala)	CH_3	1.48(d; 7.3)	18.8
		CH	3.79(m)	52.9
		COOH	b	178.5
3	Aspartate (Asp)	CH_2	2.71(dd; 17.4, 8.7)	39.6
		CH_2'	2.81(dd; 17.4, 3.7)	39.6
		CH	3.90(dd; 8.7, 3.7)	55.1
		COOH	b	179.0
4	Choline	$(CH_3)_3$	3.21(s)	56.7
		NCH_2	3.52(m)	70.1
		CH_2OH	4.07(m)	57.8
5	Citrate	CH_2	2.55(d; 15.2)	48.5
		CH_2'	2.68(d; 15.2)	48.5
		C-OH	—	78.4
		CH_2-COOH	—	181.6
		quaternary C-COOH	—	184.7
6	Creatine	CH_3	3.04(s)	39.8
		CH_2	3.93(s)	56.4
		C=NH	b	159.7
		COOH	b	177.2
7	Fatty acids and fatty acyl chains	CH_3	0.88(t; 7.2)	16.8
		CH_3-CH_2	1.28	32.6

		$(CH_2)_n$	1.30	25.7
		CH_2-CH_2-CO	1.57	27.6
		CH_2-CH_2-CH=CH	2.01	30.0
		CH_2-CH_2-CO	2.24	36.5
		CH=CH-CH_2-CH=CH	2.75	28.4
		CH=CH	5.30	131.0, 131.8
8	Formate	HCOOH	8.46(s)	172.4
9	Fumarate	CH	6.53(s)	138.1
10	Glutamate (Glu)	βCH_2	2.07(m)	30.4
		$\beta CH_2'$	2.13(m)	30.2
		γCH_2	2.35(m)	36.5
		CH	3.76(dd; 7.3, 4.7)	57.7
		COOH	b	184.3
11	Glutamine (Gln)	βCH_2	2.14(m)	29.6
		γCH_2	2.46(m)	34.0
		CH	3.78(t; 6.2)	57.4
12	Glutathione	Glu βCH_2	2.17(m)	29.3
		Glu γCH_2	2.52(m)	34.8
		Glu $\gamma CH_2'$	2.56(m)	34.8
		Cys βCH_2	2.98(dd; 14.2, 9.6)	41.7
		Cys $\beta CH_2'$	3.31(dd; 14.2, 4.4)	41.7
		Glu αCH	3.78(m)	56.7
		Cys αCH	4.76(dd)	56.0
		Gly αCH_2	b	b
13	Glycerophosphocholine (GPC)	$(CH_3)_3$	3.24(s)	57.0
		CH_2-OH	3.68(m)	65.1
		NCH_2	3.68(m)	69.1
		OCH_2 of glycerol	3.88(m)	69.6
		CH-OH	3.92(m)	72.7
		$OCH_2'$of glycerol	3.95(m)	69.6
		NCH_2CH_2	4.33(m)	62.5
14	Glycine (Gly)	CH_2	3.56(s)	44.3
		COOH	b	178.3
15	Histidine (His)	CH_2	3.18	b
		CH_2'	3.26	b

		HOOC-CH	b	b
		C=CH	7.10(s)	b
		N=CH	7.90(s)	b
		quaternary C	b	b
16	Hypoxanthine	C(2)H	8.20(s)	b
		C(7)H	8.22(s)	b
		C(9)	b	b
		C-OH	b	b
		C(5)	b	b
17	Isoleucine (ILe)	δCH_3	0.94(t; 7.4)	14.3
		βCH-CH_3	1.01(d; 7.0)	17.7
		γCH_2	1.27(m)	27.0
		γCH_2′	1.47(m)	27.0
		βCH	1.98(m)	38.9
		αCH	3.68(d; 4.0)	63.0
		COOH	b	177.0
18	Lactate	CH_3	1.33(d; 6.9)	22.8
		CH	4.11(q; 6.9)	71.3
		COOH	b	185.2
19	Leucine (Leu)	δCH_3	0.96(d; 6.1)	24.4
		δ′CH_3	0.97(d; 6.1)	25.1
		βCH_2	1.70(m)	42.4
		γCH	1.72(m)	26.5
		βCH_2′	1.74(m)	42.4
		αCH	3.74(m)	56.5
		COOH	b	117.6
20	Lysine (Lys)	γCH_2	1.45(m)	24.6
		γCH_2′	1.51(m)	24.6
		δCH_2	1.72(m)	28.5
		βCH_2	1.91(m)	32.9
		εCH_2	3.03(t; 7.5)	41.9
		CH	3.76	57.7
		COOH	b	177.5
21	Methanol	CH_3	3.36(s)	51.8
22	Methylphosphate	CH_3	3.47(d; 10.1)	54.4
23	N-acetylglucosamine(GlcNAc）	α-C1H	5.21(d)	93.3
		α-C2,5,6H	3.85 (m)	b
		α-C3H	3.76	73.5
		α-C4H	3.46	72.7
		β-C1H	4.71	97.8
		β-C2H	3.67	59.5

		β-C3H	3.53	76.7
		β-C4,5H	3.46	72.7
		NA-H	2.05 (s)	24.8
		8-CH of adenine	8.18(s)	b
		5-CH of nicotinamide	8.19(m)	b
24	Nicotinamide Adenine Dinucleotide (NAD)	2-CH of adenine	8.43(s)	b
		4-CH of nicotinamide	8.83(dd)	b
		6-CH of nicotinamide	9.14(m)	b
		2-CH of nicotinamide	9.34(s)	b
25	Phenylalanine (Phe)	CH_2	3.13(dd; 14.5, 7.8)	39.5
		CH2′	3.28(dd; 14.5, 5.2)	39.5
		N-CH	4.00(dd)	58.8
		o-CH	7.33(m)	132.0
		p-CH	7.38(m)	130.9
		m-CH	7.43(m)	132.1
		quaternary C	b	138.0
		COOH	b	175.9
26	Phosphorylcholine (PC)	$(CH_3)_3$	3.23(s)	56.8
		N-CH_2	3.59(m)	69.0
		O-CH_2	4.18(m)	60.8
27	Succinate	CH_2	2.41(s)	36.9
		COOH	b	185.1
28	Taurine	S-CH_2	3.28(t; 6.6)	50.4
		N-CH_2	3.42(t; 6.6)	37.8
29	Threonine (Thr)	CH_2	3.06(dd; 14.7, 7.7)	38.6
		CH_2'	3.19(dd)	38.6
		N-CH	3.94(dd)	59.0
		o-CH to C-OH	6.90(m)	118.3
		m-CH to C-OH	7.20(m)	133.4
		quaternary C	b	129.6
		C-OH	b	158.0
		COOH	b	177.1
30	Tryptophan (Trp)	C(8)H of indole	7.20(m)	122.3
		C(9)H of indole	7.29(m)	124.9
		C(2)H of indole	7.33(s)	128.0
		C(6)H of indole	7.55(m)	115.2
		C(7)H of indole	7.74(m)	121.3
		C(5) of indole	—	92.5
31	Tyrosine (Tyr)	CH_2	3.06(dd; 14.7, 7.7)	38.6

		CH_2'	3.19(dd)	38.6
		N-CH	3.94(dd)	59.0
		o-CH to C-OH	6.90(m)	118.3
		m-CH to C-OH	7.20(m)	133.4
		quaternary C	b	129.6
		C-OH	b	158.0
		COOH	b	177.1
32	UDP-glucose	G1-H	5.61	99.4
		C6,ring	7.96(d)	144.4
		C5,ring	5.98(d)	105.4
		C1′H,ribose	5.99(d)	91.3
		C2′3′H,ribose	4.38(m)	72.2/76.5
		C4′,ribose	4.29(m)	85.9
		C5′H,ribose	4.26/4.21(m)	67.8
		G2-H	3.9	73.1
		G6-H	3.86/3.78	63.4
		G3-H	3.77	74.7
		G4-H	3.54	74.0
		G5-H	3.47	72.0
33	UDP-Glucuronate	G1-H	5.63	99.4
		C6,ring	7.95(d)	144.5
		C1′H,ribose	6.00(d)	91.4
		C5,ring	5.974(d)	105.3
		C2′3′H,ribose	4.38(m)	86.6
		C4′H,ribose	4.29(m)	86.1
		C5′H,ribose	4.25/4.19	67.8
		G5-H	4.14(dd)	75.6
		G2-H	3.79(dd)	b
		G4-H	3.59(m)	63.2
		G3-H	3.51(dd)	72.7
34	UDP-N-acetylgalactosamine	C2′3′H,ribose	4.37(m)	76.6
		C4′H,ribose	4.29(m)	85.9
		C5′H,ribose	4.25/4.19(m)	67.8
		C1′H,ribose	5.99(d)	91.1
		C5,ring	5.97(d)	105.4
		C6,ring	7.96(d)	144.1
		NA-H	2.09(s)	24.9
		NA-C=O	b	b
		G1-H	5.56(dd)	97.5
		G2-H	4.05(m)	71.2
		G3-H	3.97(dd)	70.3
		G4-H	3.76(m)	57.4
		G5-H	3.79	63.6
		G6-H	3.78	63.6

		C6,ring	7.96(d)	144.1
		C1′H,ribose	5.99(d)	91.0
		C5,ring	5.97(d)	105.4
		C2′3′H,ribose	4.37(m)	72.2/76.5
		C4′H,ribose	4.29(m)	86.0
		C5′H,ribose	4.25/4.19(m)	67.8
		C2,ring	b	156.6
35	UDP-N-acetylglucosamine	G1-H	5.52(dd)	97.4
		G2-H	3.99(m)	56.5
		G3-H	3.82(m)	73.6
		G4-H	3.55(dd)	72.2
		G5-H	3.93	75.8
		G6-H	3.87	63.2
		NA-H	2.08(s)	24.9
		NA-C=O	b	177.4
		CH_2	3.80(dd)	b
		CH_2'	3.91(dd)	b
		C(5)H of ribose	4.14(m)	b
		C(4)H of ribose	4.24(dd)	b
36	Uridine	C(3)H of ribose	4.36(dd)	b
		C(2)H of ribose	5.92(d; 8.1)	b
		C-CH of uracil	5.91(d; 4.7)	b
		N-CH of uracil	7.89(d; 8.1)	145.6
		γCH_3	1.00(d; 7.0)	19.4
		$\gamma' CH_3$	1.04(d; 7.0)	20.7
37	Valine (Val)	βCH	2.28(m)	31.5
		αCH	3.62(d; 4.3)	63.0
		COOH	b	177.4

[#] The assignment was accomplished with the assistance of a series of two dimensional NMR spectra including ^1H–^1H COSY, ^1H–^1H TOCSY, ^1H J-resolved, ^1H–^{13}C HSQC and ^1H–^{13}C HMBC; Small signals were confirmed by standard compounds as well;

[a] Multiplicity: singlet(s), doublet(d), triplet(t), quartet(q), doublet of doublets(dd), doublet of triplets (dt), multiplet(m);

[b] The signals or the multiplicities were not determined.
